# Dynamics of membrane nanotubes coated with I-BAR

**DOI:** 10.1038/srep30054

**Published:** 2016-07-21

**Authors:** Younes F. Barooji, Andreas Rørvig-Lund, Szabolcs Semsey, S. Nader S. Reihani, Poul M. Bendix

**Affiliations:** 1 Niels Bohr Institute, University of Copenhagen, Blegdamsvej 17, 2100 Copenhagen, Denmark; 2 Department of Physics, Sharif University of Technology, Teheran 11365-9161, Iran

## Abstract

Membrane deformation is a necessary step in a number of cellular processes such as filopodia and invadopodia formation and has been shown to involve membrane shaping proteins containing membrane binding domains from the IRSp53-MIM protein family. In reconstituted membranes the membrane shaping domains can efficiently deform negatively charged membranes into tubules without any other proteins present. Here, we show that the IM domain (also called I-BAR domain) from the protein ABBA, forms semi-flexible nanotubes protruding into Giant Unilamellar lipid Vesicles (GUVs). By simultaneous quantification of tube intensity and tubular shape we find both the diameter and stiffness of the nanotubes. I-BAR decorated tubes were quantified to have a diameter of ~50 nm and exhibit no stiffening relative to protein free tubes of the same diameter. At high protein density the tubes are immobile whereas at lower density the tubes diffuse freely on the surface of the GUV. Bleaching experiments of the fluorescently tagged I-BAR confirmed that the mobility of the tubes correlates with the mobility of the I-BAR on the GUV membrane. Finally, at low density of I-BAR the protein upconcentrates within tubes protruding into the GUVs. This implies that I-BAR exhibits strong preference for negatively curved membranes.

Membrane shaping constantly takes place in cellular processes such as endocytosis^1^, cell division^2^ and in tubular protrusions like filopodia^3^,^4^,^5^ and Tunneling NanoTubes (TNT’s)^6^. These deformed and highly curved membrane structures facilitate a host of cellular processes like intercellular transport and extracellular sensing. Essential players involved in membrane deformation are proteins from the superfamily of BAR domain proteins which contain membrane binding domains with specific curvatures thus enabling the cell to create positive and negative membrane curvatures for a number of processes^7^. A family of proteins containing convex shaped dimeric I-BAR domains (also called IM domains) facilitate formation of oppositely curved membranes which are found inside tubular structures like filopodia and invadopodia^8^,^9^. The I-BAR containing protein ABBA has been associated with membrane protrusions in radial glial cells where it localizes to the inner surface in curved membrane ruffles connecting the membrane with the cortical actin cytoskeleton^10^. Deletion of ABBA in cells leads to reduced lamellipodium dynamics thus suggesting that ABBA has an important role in sensing and deformation of the ruffled membrane shape at the front of the lamellipodium. Overexpression of ABBA and other I-BAR containing proteins in cells leads to filopodia formation which shows that I-BAR domains are involved in curvature generation. The observation of membrane deformation in cells by I-BARs is directly consistent with findings that I-BAR domains have the ability to deform membranes *in vitro*, in particular negatively charged membranes containing phosphatidylserine and phosphoinositide lipids (PIP)^9^.

A number of different BAR domain proteins have been studied with respect to mechanical effects on membranes^7^ and also on curvature sensing and curvature induction^11^, but remarkably little information exists regarding the mechanical effect of I-BAR domains on membranes and how the domains are recruited to certain membrane regions. However, recently the I-BAR domain from IRSp53 was found to be highly sensitive to negative membrane curvatures^12^ and another investigation has shown that IRSp53 induces shape instability in membranes in a tension dependent manner^13^ by a similar mechanism as was found previously with N-BAR domains^14^,^15^. Also, physical characteristics, such as tube width, have been investigated by cryo-electron microscopy (cryo-EM) in fixed samples using multilamellar vesicles tubulated by different types of I-BAR domains. These experiments have revealed that different I-BAR domains produce tubules ranging from ca. 40 nm to 80 nm in diameter^9^.

The dynamics of the interaction between membrane tubes and I-BAR domains can provide interesting information regarding possible mechanical effects of the protein inside the membrane tube. Protein sorting as well as protein oligomerization and resulting stiffening of tubes has been previously reported experimentally for F-BAR domains^11^,^16^. Membrane curvature sensing by I-BAR proteins can potentially play an important role in the recruitment of proteins to high curvature regions. At higher membrane densities protein induced stiffening could play a role in stabilization of tubular membrane structures like filopodia^3^, tunneling nanotubes connecting cells^17^ and membrane ruffles at the leading edge of lamellipodia^10^. However, possible stiffening effects have so far not been studied with I-BAR proteins despite its presence in stiff cellular membrane tubes where the stiffening is often attributed to stiff polymers like F-actin inside the tube^18^. Filopodia, induced by expression of *C*. *elegans* I-BAR proteins, were reported to appear stiffer than filopodia which were formed by expression of the vertebrate I-BAR domains from MIM and IRSp53^9^. However, no quantification of filopodia stiffness was provided, and moreover, the contribution from filopodial actin cannot be neglected in cellular systems.

Experimentally, tubulation and curvature sensing by BAR domain proteins has been studied *in vitro* where the protein has been added to the outside of spherical membrane vesicles. The width of tubules formed by BAR domains binding to multilamellar lipid vesicles have been quantified by using electron microscopy^8^,^16^,^19^. Another approach is to employ optical trapping together with micropipette aspiration to pull tubes, with specific curvatures, out of GUVs which has allowed investigation of curvature sensing by various BAR domain proteins^11^,^20^. The effects of I-BAR domains on membranes remain poorly studied in reconstituted *in vitro* systems, most likely due to the natural association of the protein with the inner surface of membranes. To mimic the cellular settings the protein needs to be encapsulated in closed spherical membranes^12^,^21^. However, in the context of exploring the local effects of I-BARs, addition of I-BAR domains to the outside of GUVs can be argued to be equally relevant since the protein, at a molecular scale, essentially faces a flat GUV at the outer surface as well. Addition of I-BAR to the outside results in formation of inwards pointing tubes which cannot readily be held fixed by optical tweezers and therefore a different approach is needed to study such tubes.

To extract the isolated mechanical effect of I-BARs on membrane tubes, protruding into GUVs, it is necessary to carry out a dynamic study of the membrane nanotubes which allows extraction of tube stiffness as well as tube diameter of freely fluctuating tubes. This could answer the question concerning possible stiffening due to oligomerization of I-BAR domains inside membrane nanotubes, an effect which was reported for F-BAR domains bound on the outside of membrane nanotubes^11^,^16^.

Here we present a new strategy which can provide both the stiffness and width of nanotubes connected to a GUV, but fluctuating in three dimensions thus resembling intracellular nanotubes which are free to move in the cytoplasm, but might be connected to a larger membrane compartment. Parallel measurements of stiffness and width of unilamellar nanotubes, fluctuating in three dimensions, have to our knowledge not been investigated previously. This assay allows quantification of the physical properties of I-BAR coated nanotubes penetrating into GUVs and the effect of I-BAR can be isolated by direct comparison with protein free nanotubes of the same diameter. By performing a fluctuation analysis of the nanotubes which diffuse transiently into the focus of the microscope we extract both their stiffness and radius. We quantify the width and stiffness of I-BAR coated membrane nanotubes fluctuating inside GUVs by adding the protein to the outside of the GUVs. The tube radius is found by performing parallel intensity and fluctuation analysis of nanotubes coated with I-BAR domains at the inner surface of the tubes. By comparing the persistence length of protein coated tubes with bare tubes which are formed spontaneously in GUVs, we investigate whether I-BAR domains have an effect on the stiffness and width of the membrane nanotubes. Finally, we show how I-BAR domains exhibit strong membrane curvature sensing of negative membrane curvatures only at low membrane density of I-BAR.

## Results

**Figure 1:**
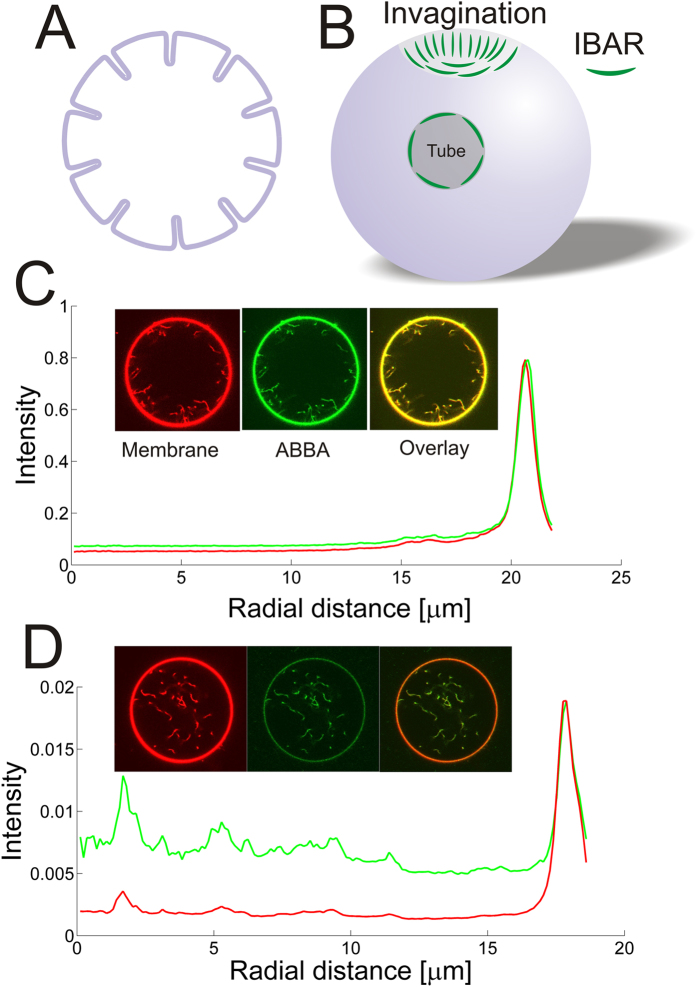
I-BAR domains from ABBA can form tubes pointing into Giant Unilamellar lipid Vesicles (GUVs) at high density or bind to existing tubes at lower density. (**A,B**) Schematic depiction showing I-BAR binding to tubes pointing inwards into the lumen of a GUV. (**C**) Incubation of a GUV with 2.3 μM I-BAR. Graph shows radial intensity plot of TR-DHPE signal (red) and YFP labeled I-BAR signal (green) from a GUV containing a number of inward pointing tubes as shown by the confocal images. (**D**) Incubation of a GUV with 290 nM I-BAR. Graph shows radial intensity of TR-DHPE signal (red) and YFP labeled I-BAR signal (green) from a GUV containing a number of inward pointing tubes as shown by the confocal images. Each intensity plotted in (**C,D**) is the average of all pixels with the same distance to the center of the GUV. The YFP intensities in (**C,D**) are normalized by the same constant. Membrane composition is DOPC:DOPS:TR-DHPE 59.7:40:0.3.

Proteins containing I-BAR domains induce shape instabilities of GUVs at a critical density and critical membrane tension^13^,^15^. At low tension I-BAR domains can induce shape changes at low protein density whereas at high tension a higher protein density is needed to deform the membrane^15^. Here we minimize the membrane tension by incubating the GUVs with the I-BAR domain from ABBA in a slightly hyperosmotic buffer (see Materials and Methods) and by minimizing adhesion of the GUVs to the glass substrate. To minimize surface adhesion and consequent increase in membrane tension we passivate the glass surface using α-casein before adding the GUVs to the imaging chamber. GUVs incubated with the I-BAR domain from ABBA exhibited membrane protrusions pointing into the lumen of the vesicle as shown in Fig. 1. Radial intensity plots for the membrane and protein are plotted in Fig. 1C,D at high and low membrane density of I-BAR, respectively. At high density the protein is nearly immobile (see Figure S1) presumably due to crowding effects whereas at low membrane density the protein recovers quickly following bleaching. The protein density used in Fig. 1D is not sufficient for tubulation, but instead the tubes were formed by a spontaneous process during the GUV-electroformation and the protein is seen to diffuse into these preformed tubes.

**Figure 2:**
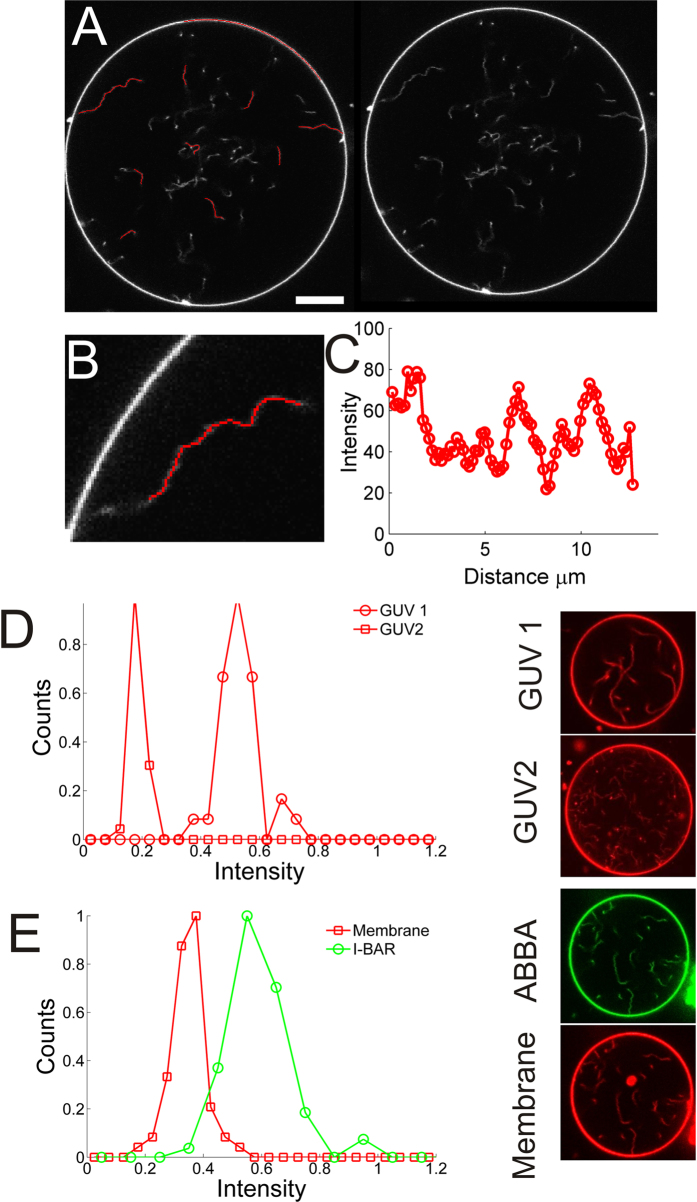
Examples of how intensities of tube segments are recorded and quantified. Tube segments transiently diffuse into focus of the confocal microscope. An adaptive filament algorithm^41^ was used to track the filaments and extract the intensity while they are in focus. By recording a number of images several different tube segments can be analyzed. (**A**) A GUV displaying a number of tube segments which are in focus (right panel). Left panel shows the fitted curves (red curves) which overlap with the tube segments which are in focus. The GUV membrane can similarly be quantified using the same strategy. Scale bar, 10 μm. (**B**) Enlarged image of a tube from (**A**) with the fitted curve as an overlay (red curve). (**C**) Intensity profile along the red curve in (**B**). The portion of the tube which is closest to the microscope focus corresponds to the maximum intensity value and is used in the following quantification of tube intensities. (**D**) Distribution of the maximum intensities from a number of tube segments from two different GUVs (images of GUV1 and GUV2 are shown to the right). The number of tube segments are *N*_seg_ = 31 (GUV1) and *N*_seg_ = 33 (GUV2). (**E**) Distribution of tube intensities from a GUV containing YFP labeled I-BAR coated tubes ([I-BAR] = 2.9 μM). The red squares represent maximum tube intensities from the membrane channel (TR-DHPE) and the green circles corresponds to the equivalent signal from the YFP labeled I-BAR (*N*_seg_ = 64). To the right are shown confocal images of the YFP labeled I-BAR (green) and membrane (red). Membrane composition is DOPC:DOPS:TR-DHPE 59.7:40:0.3.

The single tubes are imaged by confocal microscopy by imaging both the YFP tagged I-BAR as well as the Texas Red labeled lipid TR-DHPE. The measured intensity level from YFP scales linearly with membrane density of I-BAR. All intensities from YFP, presented in this work, have been normalized with the same intensity corresponding to the membrane density at which the protein was found to be immobile on the membrane. Tubes had variable lengths, but individual segments which were transiently within the quasi two-dimensional focal plane were used to quantify the membrane and protein intensity associated with the tubes as shown in Fig. 2. We analyzed the intensity of tube segments within GUVs by using an adaptive filament algorithm which adapts a curve to the shape of the filament and allows extraction of the intensity profile. Only clearly visible tube segments of length ~3 μm or more were quantified by this method (see Fig. 2A,B). The intensity along the tube was plotted and the maximum intensity corresponds to the part of the tube which is closest to the focus of the microscope (see Fig. 2C). Tubes which formed spontaneously within GUVs during the electroformation were analyzed and the maximum intensity of a number of tube segments was collected for each GUV. Histograms of tube intensities from two GUVs containing tubes of different widths are shown in Fig. 2D. The narrow distribution of tube intensities in each GUV shows that all tube diameters are similar in each GUV. The diameter of a tube connected to the GUV membrane is set by the membrane tension and the bending rigidity of the bilayer^22^ and we note that a low membrane tension is a requirement for the existence of tubes as shown in Figures S3 and S4. The membrane tension is constant for all tubes in each GUV and hence results in uniformly sized tubes, but might vary among different GUVs due to small changes in osmotic pressure and differences in adhesion to the glass substrate. This causes tubes to have different widths in different GUVs as shown by the example in Fig. 2D. As shown in Fig. 2E the intensity distribution of both the protein (green circles) and membrane (red squares) signals on tubes, from the same GUV, are normally distributed around a mean value which indicates a uniform density of protein on tubes with similar diameters.

**Figure 3:**
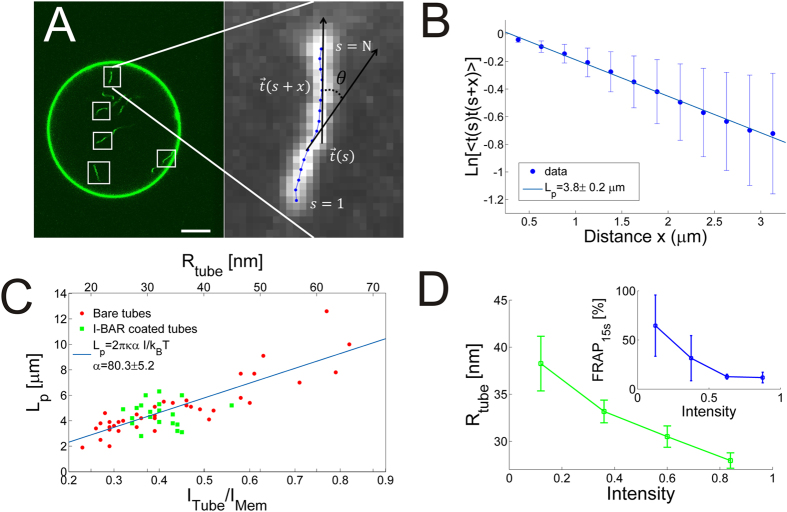
Membrane nanotubes coated with I-BAR domains have uniform widths with well-defined stiffness. (**A**) Quantification of tubular shape by finding local tangent vectors is performed on tube segments which are transiently within the focal plane of the microscope. Scale bar, 10 μm. (**B**) By analyzing the shape of <*N*_seg_> = 92 tube segments from each GUV the average correlation between tangent vectors along the tube segments is found. The slope of the graph equals the persistence length. (**C**) Persistence lengths versus the intensity ratio between the tube membrane and the GUV membrane. The corresponding radius of the nanotube is found from eq. 2 and plotted in the top axis. Each data point is obtained by analyzing, on average, the shape of 29 tube segments from each GUV. Green data points represent tubes which contain I-BAR bound to the interior of the tubes and red data points represent spontaneous tubes formed in GUVs in absence of I-BAR. Tubes from a total of 59 GUVs were analyzed and the intensity distributions had an average width of 17.0% of the mean. (**D**) Tubule width as a function of intensity of YFP labeled I-BAR. Inset shows the relative recovery after 15s, following photo bleaching of spot on the GUV membrane, as a function of intensity of YFP labeled I-BAR on the GUV membrane. The inset graph shows binned data from 21 FRAP experiments, error bars denote the standard deviation. Membrane composition DOPC:DOPS:TR-DHPE 59.7:40:0.3.

The spontaneously formed tubes in protein free GUVs were used to calibrate the stiffness and width of the tubes using a similar strategy as we used in ref. 23. The shape of the tube is tracked and the correlation of tangent vectors along the tube is quantified as shown in Fig. 3A. The correlation between tangent vectors separated by a distance, *x*, along a tube with persistence length *L*_P_ is given by^23^,^24^


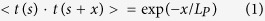


where <> denotes the average performed over the variable *s*.

Since the tubes in each GUV have similar intensity and hence similar width we can sample a number of tube segments diffusing transiently into the confocal plane of the microscope and analyze their shapes. The average correlation of a number of tube segments from a single GUV is plotted Fig. 3B. The slope of the graph in Fig. 3B gives the average persistence length of the tubes in the GUV.

Theoretically, the persistence length, *L*_p_, of a tube is linearly dependent on its radius, *R*_tube_^22^


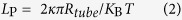


where 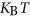 is the thermal energy, 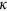 is the bending rigidity of the membrane. We used the value from ref. 13 where 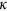 = 23 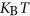 was measured for a similar lipid mixture as used here. The factor of two, in eq. 2, stems from the fact that we only detect tube segments which are within the two dimensional space of the microscope focus^25^. Therefore, by calculating the persistence length for tubes in individual GUVs we directly obtain 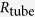. The persistence length of a number of tubes are plotted in Fig. 3C (red circles) as a function of 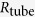 (top axis). Moreover, in the bottom axis in Fig. 3C we also plot the tube intensity originating from the membrane dye (see Fig. 2A,B), normalized by the intensity on the GUV membrane. This intensity ratio should scale linearly with the tube radius since the measured intensity from the membrane dye is a function of the tube area. The linear relation in Fig. 3C between 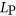 and intensity confirms that the tube stiffness is well described by eq. 2.

To investigate whether I-BAR coated tubes exhibit any stiffening relative to protein free tubes we plot the persistence length of I-BAR coated tubes together with the persistence length of protein free tubes in Fig. 3C. The tubes coated with ABBA are represented by green squares and can be seen to have similar stiffness as the protein free tubes (red circles) of the same width. However, the I-BAR coated tubes span a restricted range of radii ranging from 25 nm to 35 nm in radius. The radius of I-BAR coated tubes approaches 25 nm as the density of I-BAR on the membrane increases which is shown in Fig. 3D where tube radius is plotted versus intensity of YFP labeled I-BAR. These results indicate that tubes formed by I-BAR obtain a specific curvature dictated by the convex shape of the membrane binding surface of the I-BAR domain.

**Figure 4:**
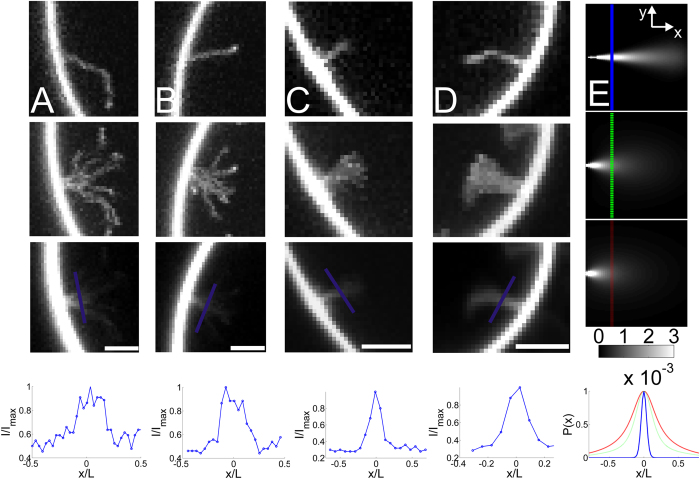
Short membrane nanotubes coated with high density of I-BAR are semi-flexible with *L*_p_ ~*L* and do not move laterally on the GUV membrane. (**A**) From top to bottom panel, (i) example of a tube fixed on the GUV membrane (ii) the corresponding maximum projection of the intensity of a series of images of the same tube (iii) the corresponding average intensity from the entire time series and (iv) line profile parallel to the GUV membrane at a distance corresponding to *L*/3 from the GUV membrane. (**B–D**) More examples of short tubes plotted in the same sequence as in (**A**). (**E**) Theoretical calculation of the probability of a fluctuating rod which is fixed at one end. Top image shows a rod which has *L*_p_/*L* = 20, in the middle image *L*_p_*/L* = 1.7 and in the bottom image *L*_p_/*L* = 0.7. *x* and *y* axes range from 0 to *L and –L to L,* respectively. The graph below the images corresponds to line profiles along the depicted lines in the three images, *L*_p_/*L* = 20 (blue), *L*_p_/*L* = 1.7 (green) and *L*_p_/*L* = 0.7 (red). All images in (**A–D**) present intensities from YFP labeled I-BAR. Membrane composition DOPC:DOPS:TR-DHPE 59.7:40:0.3. The figure shows representative examples of the (N_short_ > 100) short tubes observed in this work. All scale bars are 2 μm.

To further characterize the stiffness of the I-BAR coated tubes we used an alternative approach to measure the stiffness. By analyzing short membrane tubes fixed on the GUV membrane allowed us to image the same short tubes (*L* = 3–5 μm) for longer times, see Fig. 4. The measured positions of the tube can be compared with simulations in which tubes are modeled as stiff or semi-flexible rods with a fixed anchor point on the GUV membrane. We construct the probability distribution function for finding the tip at different locations near the GUV membrane as a function of the ratio *r* = *L*_P_/*L* as derived in ref. 26 Considering a tube fixed on the GUV membrane we define a coordinate system with *x* pointing inwards and normal to the GUV membrane, *y* is orthogonal to *x* but lies parallel within the focal plane of the microscope and finally 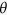 is the angle between *x* and the tangent to a point on the tube. The probability of finding a point on the tube at a location given by *x*, *y*, 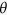 is^26^


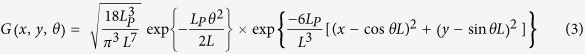


Eq. 3 gives the probability that the tip of a tube with length *L* and persistence length *L*_P_ is located at a position (*x, y, θ*). Numerically, integrating out the angle *θ* gives a two-dimensional probability distribution for the location of the tip.

In our data the whole tube is labeled and we can therefore not track the tip only. However, eq. 3 applies to any point on the tube and therefore, following integration with respect to *θ*, we integrate with respect to *L* to obtain the probability density plot for the entire tube, see Fig. 4E.

The calculations presented in Fig. 4E show that stiff rods, with a ratio of *r* = 20, fluctuate slightly with their free end (Fig. 4E, top image) whereas semi-flexible tubes with *r*~1 have a wider probability distribution, see Fig. 4E, middle and bottom images. A cross-section of the probability distribution (indicated by the lines in the images in Fig. 4) is plotted in the lowest panel of Fig. 4E. The probability density of an experimental tube can be obtained by integrating the intensity from a number of images recorded of the same fluctuating tube which is fixed on the GUV membrane. In Fig. 4A–D we show examples of short tubes connected to the GUV membrane through a fixed anchor point. The top panels show a single image of the tubes whereas the middle and bottom images represent the maximum intensity projection and average of a time series of images, respectively. The maximum intensity projection reveals all the positions that the tube has visited whereas the average of all the images in a time series is proportional to the probability density for the localization of the tube and can be compared to the analytical predictions presented in Fig. 4E. The cross sectional profiles are plotted in Fig. 4A–D and have a Full Width at Half Maximum (FWHM) of ~0.2. The theoretical predictions in Fig. 4E also reveal a FWHM of ~0.2 for tubes having *r* = 1. The persistence length thereby has approximately the same magnitude as the length of the tubes presented in Fig. 4 which means that the tubes are semi-flexible. Since, the approaches used in Figs 3 and 4 to find the persistence length of tubes give similar results, we conclude that the persistence length of I-BAR coated tubes is *L*_P_ ~4 μm at high I-BAR density at which the protein becomes immobile on the membrane.

**Figure 5:**
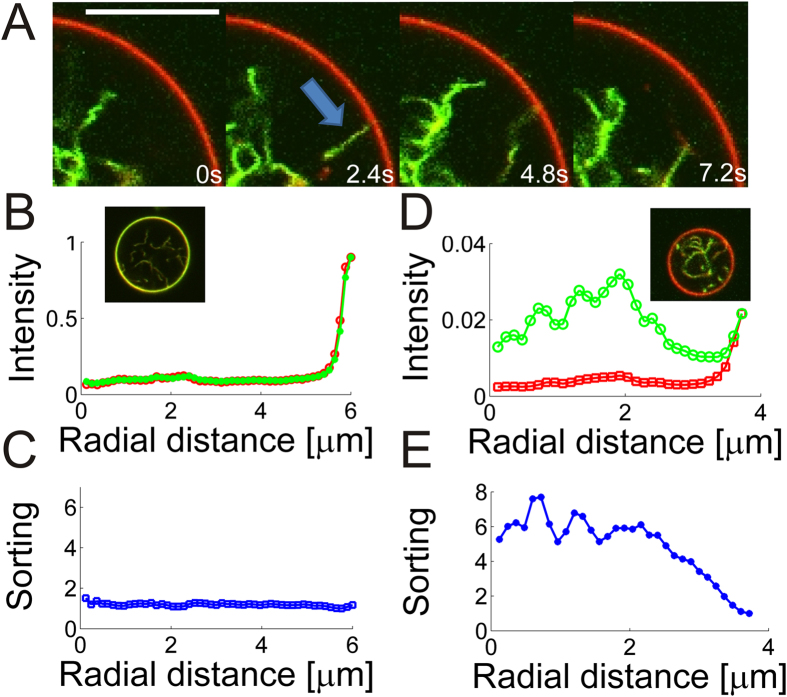
Membrane curvature sensing by I-BAR depends on protein density on the membrane. (**A**) Overlay of the membrane (red) and YFP labeled I-BAR (green) showing higher intensity of the protein inside the highly curved tube than on the nearly flat GUV membrane. The arrow shows an example of a tube connected with the GUV membrane. In subsequent images the tube disappears due to high mobility of the tube at low protein density. Protein concentration in solution is 290 nM. Scale bar, 10 μm. (**B**) Radial intensity of the membrane and YFP signal in a GUV incubated with YFP labeled I-BAR [I-BAR] = 2.3 μM. (**C**) Protein sorting as a function of distance from the center of the GUV. Sorting is defined as the ratio between the protein density on the tube and on the GUV membrane which can be calculated by using eq. 4. (**D**) Example of radial intensity at lower density of [I-BAR] = 290 nM. (**E**) Corresponding sorting of the signals presented in (**D**). Each intensity plotted in (**B,D**) is the average of all pixels with the same distance to the center of the GUV. Confocal images in (**B,D**) show the overlay of the protein (green) and membrane (red). Membrane composition DOPC:DOPS:TR-DHPE 59.7:40:0.3. Data were recorded for 15 GUVs incubated with 290 nM of YFP labeled I-BAR and all showed similar level of sorting. At micromolar concentrations of the protein we never detected any sorting (*N* = 59 GUVs).

The density of protein, however, affects the mobility of the protein as well as the mobility of the tube junctions with the GUV membrane. A high membrane density of I-BAR leads to very slow and only partial recovery of the intensity following a bleaching experiment as shown in Figure S1 and in the inset of Fig. 3D. Lowering the protein density below a certain threshold, results in high mobility of the protein which recovers after approximately 10 s, see Figure S1B and inset of Fig. 3D. A similar behavior is found for the junctions between the GUV and tubes. In absence of I-BAR or at low density of I-BAR the tubes connected to the GUV membrane move freely around the GUV (see Fig. 5A) whereas at higher density of I-BAR the tube-GUV junction becomes immobile as seen in Fig. 4 and Figure S2.

Lower membrane densities of I-BAR on the GUV membrane results in two-dimensional diffusion of I-BAR on the membrane. Interestingly, when low concentrations (290 nM) of I-BAR are added to a chamber containing GUVs, with preexisting inwards pointing tubes, we see strong preference of the protein for binding to the interior of the tubes, see Fig. 5. By quantifying the rotationally averaged radial intensity of YFP labeled I-BAR and TR-DHPE from the GUV center and out to the GUV membrane we find quite distinct relative intensities at low and high density of I-BAR, respectively. For high concentrations (>1 μM) of I-BAR the relative ratio between the protein and membrane signal is close to 1 both inside and on the GUV membrane (see Fig. 5B) and hence the density of I-BAR is the same on the GUV membrane as inside the tubes. However, at low membrane density of I-BAR we find the same ratio to be ~7 fold higher within the tubes protruding the GUV, relative to on the less curved GUV membrane (see Fig. 5D). The ratio between the protein and membrane signals in the tubes and on the GUV can be expressed as a ‘Sorting’ parameter as follows


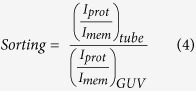


where 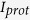 and 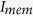 are the intensities from the YFP labeled protein and Texas Red labeled membrane, respectively. The Sorting values for the data in Fig. 5B,D are plotted in Fig. 5C,E and the data reveal that no sorting (Sorting ≈ 1) occurs in Fig. 5B whereas in Fig. 5D the density of protein is 7 times higher (Sorting ≈ 7) on the tubes than on the GUV membrane.

Since the tubes are all protruding into the GUVs these results strongly support that I-BAR domains can sense negative membrane curvatures.

Finally, we note that the range of concentrations of I-BARs used here are similar to the concentrations used in other studies where membrane deformation and membrane curvature sensing by different types of BAR domains, has been investigated in *in vitro* assays^11^,^12^,^13^,^15^,^27^,^28^.

## Discussion

I-BAR induced membrane tubes are being studied due to their similarity with protrusive structures like filopodia and invadopodia^3^,^29^. Although, filopodia-like structures can be induced by overexpression of I-BAR containing proteins^8^,^9^ the role of I-BAR domains in the formation of protrusive membrane structures remains unclear. Filopodia have been found to contain F-actin which polymerizes towards the tip and F-actin bundles have been shown to play a major role in formation and function of filopodia^3^,^18^,^30^. I-BAR containing proteins can have actin binding domains and hence could be synergistically involved in formation of filopodia together with F-actin as suggested in ref. 8. The tubulating activity of I-BAR domains from ABBA and other I-BAR domains^9^,^13^ in model membrane systems containing high density of negatively charged lipids, clearly suggests that I-BAR domains play an important role in the formation and stabilization of cellular membrane morphologies like filopodia and membrane ruffles.

Here we have shown that binding of the I-BAR domain from ABBA forms visible tubes protruding into GUVs when the I-BAR domains are added to the outer side of the GUVs at a high concentration. The tubes diffuse transiently into the focal plane of the microscope and are analyzed with respect to their intensity and shape. By exploiting the linear relation between persistence length and the width of a tube together with the fact that tube intensity scales linearly with tube radius, we can directly infer the tube width from the persistence length and intensity. At high density of protein on the membrane, the tubes are fixed to the GUV membrane and the protein exhibits minimal mobility on the membrane. Surprisingly, we find no stiffening effect on membrane tubes even at the high membrane density of I-BAR at which the protein mobility is nearly absent (see Figure S1 and inset of Fig. 3D). Previous work has shown that the F-BAR domain from Syndapin 1 induced weak stiffening of membrane tubes^11^ whereas another F-BAR protein (hFBP17)^16^ induced significant stiffening of membrane tubes by forming an oligomerized lattice around the tube. Also, N-BAR decorated membrane tubes have been shown to exhibit stiffening with a persistence length of 9.1 μm^16^ which is quite significant considering the thin tubules formed by N-BAR (~20 nm in diameter)^16^. However, in all studies which reported persistence length measurements of membrane nanotubes, the tube width and possible multilamellarity, was not measured despite the linear dependence between persistence length and tube radius (see. Eq. 2).

Comparing the measured stiffness of I-BAR coated tubes with protein free tubes we see that I-BAR produces tubes of a certain diameter of ~50 nm which is consistent with previous data from ref. 9 where diameters were measured using electron microscopy of multilamellar lipid vesicles which had been mixed with I-BAR. This suggests that membranes adapt to the molecular curvature of I-BAR domains. Moreover, since many preexisting tubes are found in the GUVs this means that I-BAR either constricts or expands preexisting tubes if they are wider or narrower than 50 nm; a similar effect was recently measured for the I-BAR from IRSp53^12^ in a different assay.

The importance of considering the lamellarity and width of the tubes is evident from the known dependence of tube width and lamellarity on tube stiffness^23^. Tubes having several bilayers with a small separation between each bilayer may appear unilamellar in bright field microscopy which has been used for analyzing the stiffness of tubes^16^. However, fluorescence microscopy of tubes can be used for both size calibration and to detect possible multilamellarity^23^.

The persistence lengths presented in Fig. 3C are plotted versus radius and therefore the effect of the protein on the tube stiffness can be isolated from the effect of tube width. The experimental settings mimic the environment that tubes experience in cells where tubes are diffusing in three dimensions, but with one end fixed to some larger membrane structure. Consequently, the tubes studied here are constantly undergoing thermal motion which causes the protein conformation on the tube to be constantly exposed to stress and potential rearrangements through bending fluctuations leading to high local membrane curvatures. The absence of any stiffening effects of the protein on the tube is surprising considering the lack of recovery of the protein following bleaching (Fig. 3D, inset and Figure S1). Previous work has also shown very slow recovery and only partial recovery following bleaching of fluorescently labeled F-BAR^27^ or I-BAR^28^ which indicated protein oligomerization. The low protein mobility together with the absence of stiffening observed here could suggest that I-BARs interact through flexible linkages.

Membrane curvature sensing experiments are typically performed using optical tweezers for pulling out high curvature membrane tubes from a GUV which is exposed to a solution containing the protein. Recently, a different approach was used where the I-BAR domain from the IRSp53 protein was first encapsulated within the lumen of GUVs followed by extrusion of tubes by an optical trap. This allowed the I-BAR domains to sense membrane curvature from inside of the GUV^12^. The I-BAR domains from ABBA used here could not be encapsulated using standard electro-formation protocols and therefore we measured the membrane curvature sensing by I-BAR inside membrane tubes protruding into the GUVs thus allowing the protein to be added to the outside of the GUVs. These results revealed a strong preference of I-BAR for the negatively curved membrane inside tubes which are unrestricted by optical tweezers (see Fig. 5).

The mechanism of membrane curvature sensing by BAR and I-BAR domains has been ascribed to both the geometry of the dimer^31^ as well to amphipathic helices which can insert into a curved membrane. The I-BAR domain from MIM (MIM-IMD) was found to sense positive curvatures on small lipid vesicles and consequently it was concluded that the I-BAR domain in MIM is not critical in membrane curvature sensing^32^. However, here we find that the closely related I-BAR domain from ABBA does indeed sense negative membrane curvature. The I-BAR domain from ABBA also contains a membrane inserting amphipathic helix which could assist binding to flat or positively curved membranes, but is unlikely to contribute to curvature sensing inside negatively curved nanotubes. Therefore, we expect the membrane curvature sensing presented in Fig. 5 to be induced by the shape of the convex binding side of I-BAR. This is consistent with the negative membrane curvature sensing reported for IRSp53 I-BAR domain which binds to membranes electrostatically without insertion of an amphipathic helix^12^. In conclusion, these results indicate that the I-BAR domain of ABBA could be an important regulator of protein density by sensing membrane curvature in e.g. membrane ruffles found in glial cells.

Membrane protein density and membrane tension have recently been proposed as master regulators of a number of cellular processes which involve a change in membrane morphology and membrane area^15^,^33^,^34^,^35^. The membrane shaping process is naturally more energy expensive at high membrane tension and hence the deformation carried out by proteins is counteracted by high tension^13^,^14^,^15^. N-BAR domains are well known to be curvature sensitive at low density whereas at high density they facilitate membrane deformation. Recently, it was shown how membrane tension could control the shape instability of GUVs induced by N-BAR domain of endophilin^15^ as well as for the I-BAR domain from IRSp53^13^. A transition density for shape instability was quantified as a function of tension thus clearly demonstrating the interplay between tension and protein density in the membrane shaping function of BAR domains. Here, we observe a similar effect of tension and concentration on shape instability, but with the I-BAR domain from ABBA. GUVs tubulate efficiently under low tension, as shown in Supplementary Figure S3 by exposing GUVs to a solution with high osmolarity delivered by a micropipette. Also, we find that existing tubes can be reversibly retracted into the GUV membrane after varying the membrane tension by successively aspirating a GUV into a micropipette, as presented in Figure S4.

The spontaneous tubes observed in our experiments in absence of I-BAR appear consistently in a fraction of the GUVs and their tube radius varies according to the data in Fig. 3C (circles). These tubes could result from the fact that experiments are carried out in a hyperosmotic buffer. Also, we note that highly charged lipids are exquisitely sensitive to local pH variations and these tubes could be due to lipid flip-flop between the inner and outer leaflet of the GUV caused by slight changes in pH across the membrane. A pH gradient has previously been shown to induce membrane deformations in GUVs containing high percentage of charged lipids^36^. Finally, we note that the electroformation process is not understood despite its wide use for making GUVs. A compositional asymmetry between the leaflets caused by some artefact from the electroformation process cannot be ruled out.

The quantitative assay developed here to investigate the properties of I-BAR coated membrane nanotubes was tested on a simple binary lipid mixture. Tube formation by IRSp53 was recently shown to be significantly enhanced by including 5% of phosphatidylinositol bisphosphate (PI(4,5)P_2_) in the GUV membrane compared with GUV membranes containing no PI(4,5)P_2_, but containing an overall identical lipid charge density^13^. Also, I-BARs have been shown to cluster PI(4,5)P_2_ lipids and reduce their diffusion^28^ which indicates strong binding between the I-BAR and the PI(4,5)P_2_ lipids. In future work the physical properties of I-BAR coated tubes should be investigated with a composition containing other important lipids like DOPE, cholesterol as well as phosphoinositide lipids.

## Conclusion

By employing a new method for quantifying the size and properties of freely fluctuating lipid nanotubes we have here shown how I-BARs deform membranes into semi-flexible tubes that have a narrow size distribution. The diameter of the tubes approaches 50 nm at high membrane density of I-BARs. No tube stiffening could be attributed to the binding of I-BAR and tubes formed by I-BAR were found to be semi-flexible (*L*_p_~4 μm) in accordance with the stiffness expected for tubes of similar diameter. I-BAR was found to be sensitive to membrane curvature with a ~7 fold increase in density inside high curvature nanotubes relative to the density on the nearly flat GUV membrane. The coupling of cellular shape, lateral membrane tension and membrane mechanics is emerging as the main regulator of cellular function, but needs to be further explored since it seems to involve a complicated interplay between membrane tension, protein activity and also lateral protein density. Here we have demonstrated a new strategy for quantifying the stiffness and width of unilamellar lipid nanotubes in three dimensions. This assay can be further used to reveal physical properties of membrane nanotubes interacting with other proteins or for investigating the stiffness of nanotubes with different lipid compositions in the vicinity of critical phase transition temperatures^37^.

## Materials and Methods

### Microscopy

Confocal fluorescence microscope (Leica SP5) equipped with a water immersion objective (Leica, PL APO NA: 1.2, 63×) and photomultiplier tubes for detection was used to record all fluorescent images. Excitation of YFP was performed at λ = 488 nm and TR-DHPE was excited at λ = 594 nm and the emitted light was collected at 495–570 nm and 612–744 nm for the two fluorophores, respectively. FRAP measurements were performed by choosing a region on the GUV membrane and briefly exposing it to high laser power from the 488 nm laser line. Since membrane nanotubes were acquired using confocal scanning microscopy which is a sequential scanning of a two dimensional space we carried out a control to check if sequential acquisition of the area affected the quantification of the persistence lengths. As shown in Figure S5 we measure similar persistence lengths using a sequential scanning technique as when using wide field excitation with camera detection. This can be rationalized by considering the time it takes to scan a tube segment of 5–10 μm in length: with a scanning frequency of 400Hz and pixel size of 160 nm it takes less than 100 ms to scan the largest tube segments in our data. The diffusion length scale during the same time interval of a tube segment having a diameter of 50 nm and length of 10 μm is <200 nm^38^,^39^ and hence this is on the same scale as our pixel width. Since the time scale of bending of these nanotubes is on the order of 1s^38^ we conclude that persistence lengths can be accurately quantified by sequential confocal scanning microscopy which requires ~100 ms to record the shape of a nanotube segment. Since the imaging depth of the confocal microscope is ~0.5 μm we can obtain proper two dimensional images of the tube segments; the tubes are stiff on a 0.5 μm length scale and hence if they bend in the axial direction they will disappear from the imaging plane.

### Electroformation

GUVs were formed by using a Nanion Vesicle Prep Pro electroformation device; 50 nmoles (50·10^−9 ^moles) of lipid mixture dissolved in 25 μL of chloroform was dropcast on a conducting ITO-coated glass slide and placed in a vacuum chamber for ~1 h to remove residual chloroform solvent. To facilitate hydration 275 μL of a 333 mM sucrose solution was then added to the dry lipid film and confined by a *d* = 18 mm O-ring. We found that adding 1mM of TRIZMA-base to the hydration medium resulted in higher number of spontaneous tubes protruding into the GUVs. This method was used for forming the protein free tubes in Fig. 3C. The chamber was sandwiched with a second ITO-slide. Unless stated otherwise all GUVs were prepared from a lipid/dye mixture composed of DOPC (1,2-dioleoyl-sn-glycero-3-phosphocholine, Avanti polar lipids), DOPS lipids (1,2-dioleoyl-sn-glycero-3-phosphoserine, Avanti Polar Lipids) and 1,2-dihexadecanoyl-sn-glycero-3-phosphoethanolamine-Texas Red was purchased from Invitrogen. Unless noted otherwise the lipids were mixed with the following stoichiometry: DOPC:DOPS:TR-DHPE 59.7:40:0.3. After 2 hours of standard electroformation (10 Hz, 1 V, 55 °C) a high yield of GUVs could be transferred to an eppendorf tube and used immediately or stored overnight at 4 °C in the dark.

### Sample preparation

Experiments were conducted on cover slips with a thickness of 170 μm which were cleaned in repeated rounds in detergent and alcohol baths under ultrasonication. The glass was treated with 2 g/L α-casein (from Sigma Aldrich) suspended in 20 mM Tris and 2mM EDTA by incubating for 30 min followed by thorough rinsing with a phosphate buffered (pH 7.2) solution containing 50 mM NaCl and 295 mM sucrose. The circular glass was inserted into a chamber with a teflon inset which confines the solution. The passivation of the surface minimized the adhesion of the vesicles to the glass. GUVs were mixed with protein at the desired protein concentration and incubated for 30 min in a 1 mL plastic tube placed on a rotating wheel to prevent sedimentation of the GUVs. The GUVs were suspended in a PBS buffer containing 50 mM NaCl and 295mM sucrose and hence the solution had a higher osmolarity than the solution inside the GUVs.

### Micropipette experiments

Glass micropipettes were fabricated using a micropipette puller (P-97 Flaming/Brown Micropipette Puller, Sutter Instruments) and microforged to a diameter of 8 μm using Micro Forge MF-900, Narishige co. ltd Japan. The pipettes contained a solid filament on the inner surface of the glass to facilitate easy back-filling of the pipette with either protein or buffer solution. Micropipettes were controlled using a standard micromanipulator (Narishige co. ltd Japan) attached to a two dimensional piezo stage (PI 731.20, Physik Instrumente, Germany) to facilitate precise positioning of the pipette.

### Protein expression and purification

The plasmids encoding the hexahistidine-tagged I-BAR domains were a kind gift from Pekka Lappalainen (University of Helsinki). The region containing the T7 promoter and the ABBA I-BAR domain was PCR amplified using the primer 5′-TTTTGAATTCTAATACGACTCACTATAGGGAATTGT-3′ and the primer 5′-TCTCACCGGTAGAACCCTTCAGGTCTTTGATCACCT-3′. The amplified DNA fragment was digested with *Eco*RI and *Age*I and inserted between the same sites of plasmid pSEM3073^40^. The resulting plasmid encoded the I-BAR domain fused to an N-terminal His-tag and a C-terminal YFP-tag. The fusion protein was expressed in *Escherichia coli* BL21 (DE3) cells. Harvested cells were resuspended in Lysis I buffer (10 mM sodium phosphate, pH 8.0, 0.5 mg/ml lysozyme) and stored on ice for 30 min. Equal volume of Lysis II buffer (10 mM sodium phosphate, pH 8.0, 2 M NaCl, 20% glycerol, 0.1% Triton X-100) was added and incubated for 30 min on ice. The cell debris was removed by centrifugation. Addition of 3% Ni-NTA slurry (Qiagen) to the solution was followed by 1 h incubation at 4 °C. A Poly-Prep Chromatography Column (BIO-RAD) was used to collect the protein bound to Ni-NTA agarose from the mixture. Twenty column volumes of washing buffer (10 mM sodium phosphate, pH 8.0, 1 M NaCl, and 10% glycerol) was allowed to flow through the column. Proteins were eluted by four column volumes of elution buffer (10 mM sodium phosphate, pH 8.0, 1 M NaCl, 50% glycerol, 250 mM imidazole). The imidazole and the residual Triton X-100 was removed by dialysis, using the final storage buffer (20 mM HEPES, 150 mM NaCl, and 1 mM TCEP (Tris (2-carboxyethyl) phosphine). The purified protein was aliquoted and stored at −80 °C.

### Data analysis

Images were recorded using Leica Application Suite and exported images were analyzed using custom made software in Matlab (The MathWorks, Inc., Natick, Massachusetts, United States) and using an ImageJ plugin called Jfilament^41^ for extracting the intensity of tube segments. Tube persistence lengths were calculated based on the method described in ref. 23. Intensities of tube segments and the GUV membrane were found by using an adaptive filament algorithm^41^ which fits a curve to the maximum intensity along a segment. Quantification of protein sorting in Fig. 5 was performed using Matlab by calculating the radial, rotationally averaged, intensity from the center of the GUV to the GUV membrane for both the membrane and protein channel. The center of the GUV was found by fitting a circle to the GUV. The ratio between the intensities directly gives the Sorting according to eq. 4. Intensities from YFP labeled I-BAR in Fig. 3C,D were quantified using the Jfilament adaptive algorithm^41^ to fit a curve to the relevant GUV membrane and extract the average GUV intensity. Normalized YFP - intensities used throughout the paper refer to the same normalization constant which was the highest YFP intensity measured on the GUV membranes. In the sorting experiments in Figs 1D and 5D we used higher laser excitation to visualize the low density of YFP labeled I-BAR. The relative ratio between the excitation powers used in the paper was quantified by measuring reflection signals from the glass/water interface at different laser powers and this allowed us to use the same normalization of the YFP signal in all figures.

## Additional Information

**How to cite this article**: Barooji, Y. F. *et al*. Dynamics of membrane nanotubes coated with I-BAR. *Sci. Rep.* **6**, 30054; doi: 10.1038/srep30054 (2016).

## Supplementary Material

Supplementary Information
