# Supplementary material for: Dynamics of membrane nanotubes coated with I-BAR
Source: Sci Rep. 2016 Jul 21;6:30054. doi: 10.1038/srep30054 (PMC4956757; doi:10.1038/srep30054)
Supplement: Supplementary Information [file srep30054-s1.pdf]

Supplementary Information for:

Dynamics of membrane nanotubes coated with I-BAR

Younes F. Barooji[1], Andreas Rørvig-Lund[1], S. Nader S. Reihani[2], Szabolcs Semsey[1], Poul M. Bendix[1]*.

[1]Niels Bohr Institute, University of Copenhagen, Blegdamsvej 17, 2100 Copenhagen, Denmark

[2]Department of Physics, Sharif University of Technology, Teheran 11365-9161, Iran

*Corresponding author: Bendix@nbi.dk

Tubes are immobile at high membrane densities of I-BAR

We imaged GUVs at the onset of tubulation as presented in Figure S2. The short length of the tubes in such experiments (< 5µm) allows us to measure the entire length of the tubes in a single image. The tubes are clearly stiff at a few µm length scale as found by plotting angular intensity profiles at different distances away from the GUV membrane (yellow, red and green lines in Figure S2A,B). The correlation coefficient between angular intensity profiles, taken at different distances R from the GUV membrane, with the intensity profile closest the GUV membrane (yellow) reveal a strong correlation at short distances whereas at ca. 3 µm the correlation decays to zero, see Figure S2C. Although, such analysis does not directly give the persistence length of tubes it does, however, reveal that tubes are stiff at a micrometer length scale and do not move laterally when the membrane density of I-BAR is high.

These short and stiff rod-like tubes stay connected to the same point on the GUV membrane for at least 30 seconds as shown in Figure S2D,E. The image in Figure S2D is a single confocal image whereas the image in Figure S2E is an average of 30 images acquired with a time resolution of delta t = 2.2s. Interestingly, the tubes can still clearly be distinguished after averaging 30 images taken at different times which shows that the tubes are not undergoing significant bending or lateral diffusion on the surface of the GUV. By correlating an angular intensity profile from the first image with the subsequent images we find that the correlation coefficient between intensity profiles, calculated using the 'corr2' matlab function, was 0.5 even after 65s which demonstrates that the tubes are relatively immobile at this time scale (Figure S2F). However, we note that extending the observation period results in slight drift of the chamber which de-correlates the signals as does flow in the chamber which causes some movement of the tubes.

The thermally driven bending of stiff tubes connected to a GUV membrane can be modeled as rods fixed on a flat wall (see Figure S2G) and can be characterized by a length parameter along the tube, 's', and the angle to the direction orthogonal to the GUV membrane θ. For the tubes in Figure S2E the persistence length is clearly longer than the length of the tubes and the energy can therefore written as

$$E_{arc} = \frac{\kappa_f \theta^2}{2s} \qquad \text{eq. S1}$$

where θ and s are defined in Figure S2G and $\kappa_f = \kappa\pi R_o$is the stiffness of the membrane tube having membrane bending rigidity κ[22]. The persistence length of the tubes is given by $L_{\mathrm{P}} = \kappa_f / K_{\mathrm{B}} T$.

Tubulation is highly sensitive on local concentration of I-BAR

The dynamic behavior of I-BAR domain induced deformation can be studied by locally adjusting the protein concentration. Local and transient changes in concentrations of I-BAR proteins in cells could be a way for cells to induce membrane tubules like filopodia in concert with other proteins like actin and actin binding proteins.

We dynamically change the concentration of I-BAR around GUVs by varying the flow out of a micropipette placed near the GUV. Importantly, we inject a highly concentrated solution of I-BAR into a chamber containing GUVs thus exposing the GUVs to high concentrations of the protein as well as a hypertonic solution, see Figure S3A. For the experiment shown in Figure S3, the high salt and glycerol concentration used in the protein purification was not removed which makes the solution highly hypertonic compared to the interior GUV solution. The solution in the pipette contains 36 µM of I-BAR in a solution containing 1M NaCl and 50% glycerol, but the solution concentration away from the pipette is quickly diluted and varies spatially. The osmotic shock induced by the concentrated solution results in deformation and shrinkage of the GUVs as seen for protein free GUVs exposed to the same solution conditions, as shown in Figure S3F. However, protein binding to the GUV resulted in formation of inward pointing tubes with uniform intensities, see Supplementary Figure S3. A variable flow of I-BAR through the micropipette led to a time dependent protein concentration around the GUV with a spatial intensity gradient pointing towards the pipette as shown in Figure S3B. An increase in protein concentration in solution led to higher densities of I-BAR on the GUV membrane followed by inward tubulation of the membrane, see Figure S3C. Interestingly, reversing the flow led to disappearance of the tubes within seconds and tubes reappeared after injection of more protein, see Figure S3C. The location of the tubes correlated well with the intensity outside the GUV as shown by the angular intensity profiles in Figure S3D,E. The colored circles in Figure S3D outside (red circle) and inside (green, blue and yellow circles) the GUV membrane (gray) correspond to the angular intensity profiles plotted in Figure S3E. Clearly, the tube density is highest along the direction of the intensity gradient outside the GUV and hence tubulation depends on the local concentration of the injected solution.

In experiments where the injection of the protein was fast the tubes appeared randomly within the GUV as shown in Figure S3. In such experiments the entire GUV membrane is rapidly decorated with protein. Notably the size of the GUVs reduced concomitantly with appearance of tubes which can be seen by plotting the external and internal intensities with the change in GUV radius in Figure S3G. The reduction in the size of the GUV results from a transformation of the GUV membrane into tubes. Reversion of the tubes into the GUV membrane follows when protein concentration and osmotic pressure are reduced. A control experiment with injection of the same buffer (same osmotic pressure) but no I-BAR resulted in a variety of membrane deformations like pearling, tubes and larger scale deformation of the GUV shape as shown in Figure S3F.

Tubulation is regulated by membrane tension

Cell membrane tension has been suggested as an important regulator of membrane morphology in important processes like endocytosis and filopodia formation[33, 34]. Membrane invaginations like caveolae have been suggested to buffer membrane area and to be sensitive to changes in membrane tension. Membrane protrusions are efficiently counteracted by membrane tension and the energy needed to form and sustain such deformations of the cell membrane can be delivered by polymerization of actin and binding of membrane shaping proteins containing I-BAR domains. Formation of the tubes costs energy both in terms of membrane bending as well as energy for stretching the membrane during tubular protrusion into the GUVs.

To further test if the tension induced by the osmotic shock in Figure S3 could influence the tube formation we use a standard micropipette aspiration assay for changing the membrane tension of I-BAR coated GUVs (see Figure S4A). A GUV displaying tubes formed by incubation with I-BAR from ABBA at a constant concentration (3 µM) was gradually aspirated into a micropipette by increasing the suction pressure as shown in Figure S4. The membrane tension increases with the length of the aspirated area inside the pipette and the tubes, which were initially present, gradually become shorter and finally disappear. The integrated intensity inside the GUV is plotted versus time in Figure S4C and clearly shows that the tube intensity decreases over time as the aspirated length is increased.

Interestingly, after release of the pressure in the micropipette and hence lowering of the membrane tension the tubes could be found to reappear although with shorter tube length as shown in the experiment presented in Figure S4D. A total number of 16 GUVs were aspirated and tubes were found to disappear in all aspiration experiments.

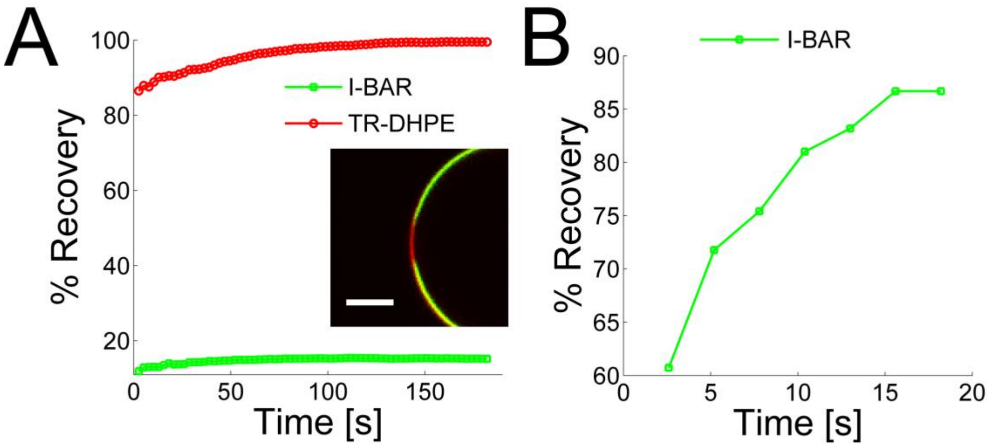

Figure S1 I-BAR mobility on the GUV membrane depends on the protein density. (A) Fluorescence recovery after bleaching of ABBA-IMD-YFP (green) and TR-DHPE (red) on the GUV membrane. Normalized intensity of YFP-I-BAR is I = 0.52. Image shows an overlay between the TR and YFP signal just after bleaching. Scale bar, 10 µm. (B) Fluorescence recovery after bleaching of ABBA-IMD-YFP (green) on the GUV membrane at lower I-BAR density on the membrane. Normalized intensity of ABBA-IMD-YFP is I = 0.27.

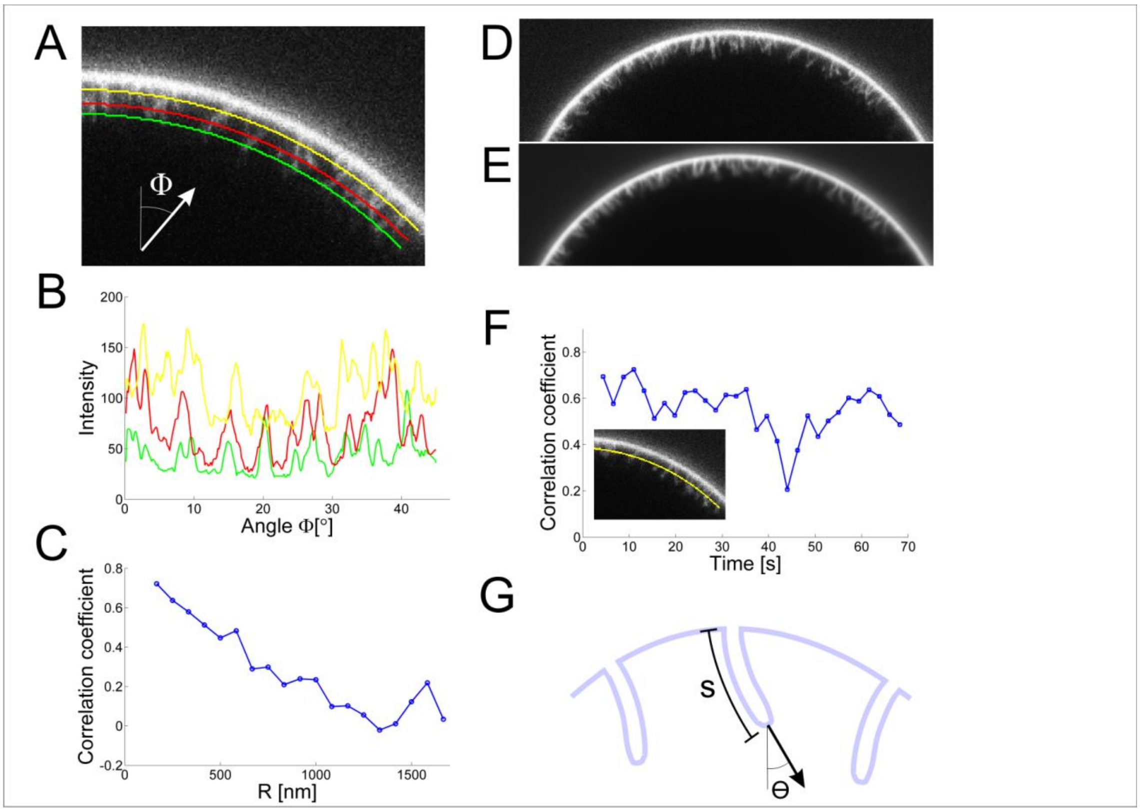

Figure S2 I-BAR coated membrane tubes form as immobile rods attached to the GUV membrane at micromolar concentrations. (A) Short tubes formed from a GUV with membrane composition DOPC:DOPS:TR-DHPE = 60:39.7:0.3. (B) Intensity profiles across the angular direction shown in (A). The three graphs correspond to the locations shown in (A). (C) Correlation coefficient between intensity profiles at different distances away from the GUV membrane. Each point represents the correlation between the intensity profile, at the given distance R from the GUV membrane, with the intensity profile just inside the GUV membrane. (D) A larger portion of the same GUV showing a number of short tubes protruding into the GUV. (E) Overlay of 30 images acquired at the same location as in (D) reveals that tubes do not move significantly at the base whereas the tip of the tube fluctuates. (F) Correlation coefficients between intensity profile inside the GUV membrane as a function of time. Each point represents the correlation of the intensity profile at t = 0 s with the intensity profile at a given time t. (G) A schematic of the bending of short and stiff tubes with the corresponding angles and parametrization by the variable 's'.

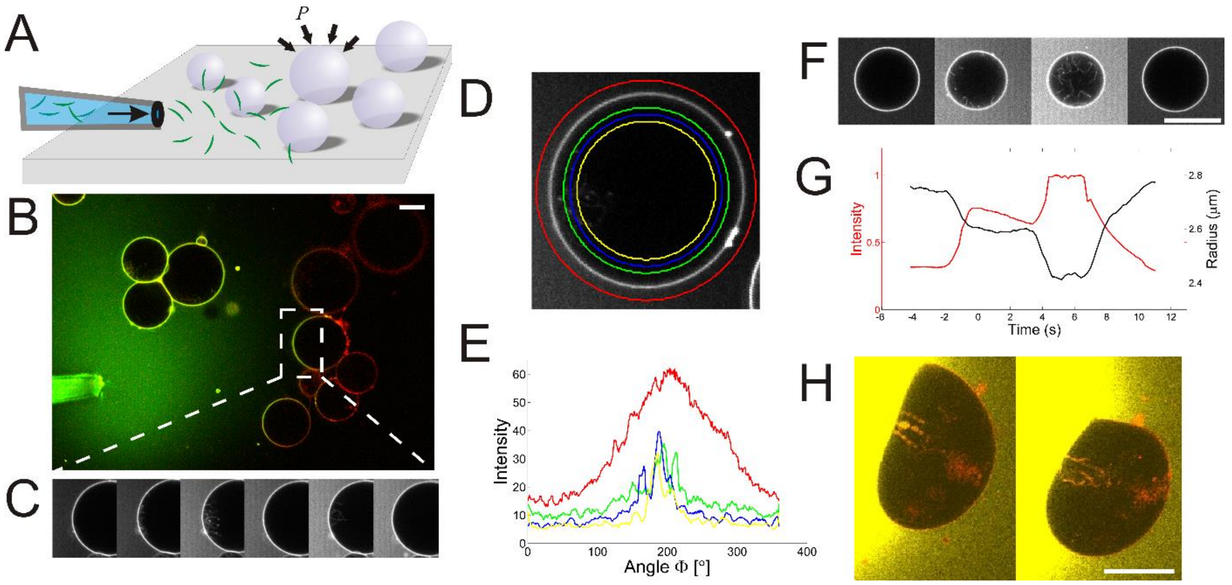

Figure S3 Dynamic behavior of membrane tubulation in GUVs exposed to I-BAR. (A) Schematics of the microinjection of I-BAR into a microscope chamber containing a number of GUVs. The protein buffer contains a higher osmolarity than the internal solution within the GUV which results in compression of the GUVs (arrows) and hence a lowering of the membrane tension. (B) Injection of I-BAR into the microscope chamber results in immediate binding to the charged GUVs (DOPC:DOPS:TR-DHPE 59:40:1) and subsequent formation of tubes coated with I-BAR. Scale bar, 10 µm. (C) Reversible tube formation triggered by varying the osmolarity and protein concentration by controlling the injection velocity. (D) A GUV showing local tube formation. (E) Angular intensity profiles corresponding to the circles in (D). The red line is the intensity outside the GUV and hence represents the local protein concentration. (F) Shrinkage and tubulation in a GUV exposed to a hyperosmotic buffer containing 36 µM of I-BAR. The injection velocity is higher than in (B-D) which results in more uniform tubulation. Scale bar, 5 µm. (G) Change in the radius of the GUV (black curve) versus time plotted together with the intensity of ABBA-IMD-YFP outside the GUV (red curve). (H) Control with the buffer used in (B,C) but with no I-BAR. Trace amount of Dextran 50kD labeled with Alexa 647 was added to the injection solution. The experiment shows that severe deformations, inward tubulations and pearlings can form even in absence of I-BAR due to osmotic pressure induced by the hyperosmotic solution. The osmotic pressure induces shape changes of the whole GUV and causes both pearling and tubes inside the GUV whereas I-BAR results in regular tubes of uniform intensity. Scale bar, 30 µm.

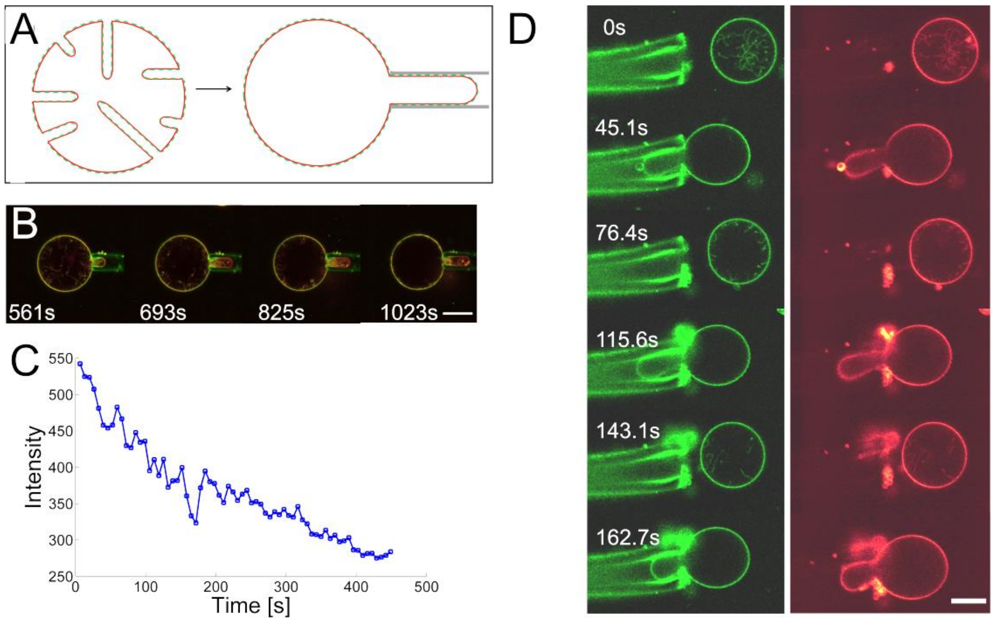

Figure S4 Membrane tubulation by I-BAR from ABBA can be regulated by membrane tension. (A) Schematic depiction of the experimental approach. A GUV containing visible tubes is aspirated into a micropipette which results in an increase in membrane tension and consequently reduction in the number and length of tubes. (B) Images from a typical experiment. Note how the tubes disappear as the aspirated length inside the micropipette increases from left to right. Images are overlays of the membrane and protein signal. (C) Integrated intensity within the GUV for the experiment in (B) as a function of time. (D) Reversible tubulation after two successive aspirations of the same GUV. Green images represent the signal from ABBA-IMD-YFP and the red color is the signal from the membrane dye TR-DHPE. The numbers in the images indicate the time in seconds. Scale bars in (B) and (D), 10 µm. A total number of 16 aspirations were performed and the tubes were found to disappear in all experiments.

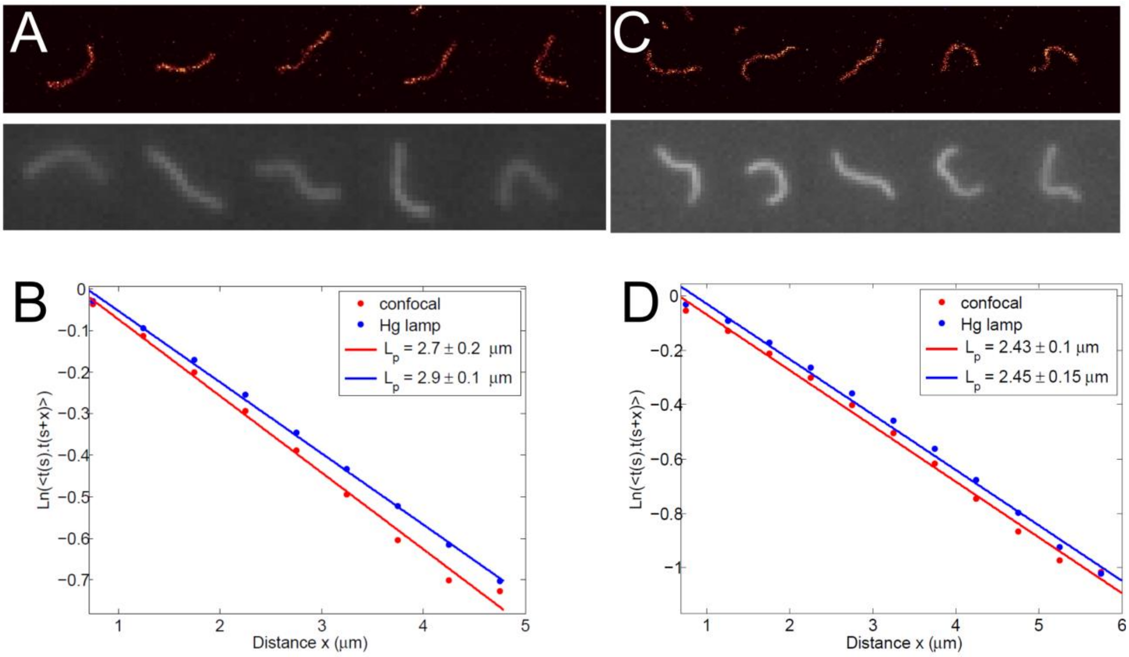

Figure S5 Control showing that imaging tubes by confocal scanning or by wide field illumination results in similar persistence lengths. (A,C) Two examples of fluctuating tubes recorded in a quasi-2D chamber in which the tubes are confined between two glass coverslips. Top row shows the tube recorded by confocal microscopy using scanning laser and a photomultiplier tube for sequential detection. Bottom row shows images recorded by exiting the fluorophores by a fluorescent lamp and detection of the emission by a camera. (B,D) Corresponding tangent correlations for the two examples in (A) and (C). The slope of the graphs gives the persistence lengths for the tubes when detected by confocal scanning (red points) or by camera (blue points). The corresponding fits result in similar persistence lengths for the two methods.
